# Molecular, spectroscopic and thermochemical characterization of C_2_Cl_3_, C_2_F_3_ and C_2_Br_3_ radicals and related species

**DOI:** 10.1098/rsos.240448

**Published:** 2024-11-20

**Authors:** María Liz Ferreira, Franco Ignacio Dubois, María Eugenia Tucceri, María Paula Badenes

**Affiliations:** ^1^ Facultad de Ciencias Exactas, Departamento de Química, Instituto de Investigaciones Fisicoquímicas Teóricas y Aplicadas (INIFTA), Universidad Nacional de La Plata, CCT La Plata-CONICET, Casilla de Correo 16, Sucursal 4, (1900), La Plata, Argentina; ^2^ Facultad de Ciencias Exactas y Naturales (FACEN), Departamento de Química, Universidad Nacional de Asunción, Campus Universitario, San Lorenzo, Paraguay; ^3^ Facultad de Ciencias Exactas, Departamento de Química, Centro de Investigación y Desarrollo en Ciencias Aplicadas 'Dr. Jorge J. Ronco' (CINDECA), Universidad Nacional de La Plata, CCT La Plata-CONICET, La Plata, Argentina

**Keywords:** quantum-chemical calculations, standard enthalpy of formation, UV absorption spectra, trifluoroethenyl radical, trichloroethenyl radical, tribromoethenyl radical

## Abstract

This work reports a detailed theoretical study of the molecular parameters, harmonic vibrational frequencies, UV absorption spectra and standard enthalpies of formation for the radicals C_2_X_3_ (with X = F, Cl and Br) and a comparison with the corresponding determinations for the rest of the members of the family C_2_X_*n*_ (with *n* = 2-4). Molecular properties were calculated using different levels of theory: density functional theory employing the B3LYP, X3LYP, BMK, M06-2X and M08-HX functionals combined with the basis sets 6–311++G(3df,3pd) and aug-cc-pVTZ, and the *ab initio* composite models G3B3 and G4. Structural and spectroscopic characterization of the C_2_F_3_, C_2_Cl_3_ and C_2_Br_3_ radicals, along with the estimation of the enthalpies of formation of C_2_F_3_ and C_2_Cl_3_, were derived here for the first time, to our knowledge. In particular, values of −220.9 ± 2.9, 230.8 ± 3.8 and 375.4 ± 5.9 kJ mol^−1^ were computed for enthalpies of formation of C_2_F_3_, C_2_Cl_3_ and C_2_Br_3_, respectively. Additionally, enthalpies of formation for related closed-shell molecules were obtained with less uncertainty compared to those found in the literature. The recommended values of −669.6 ± 3.8, −23.0 ± 4.6 and 155.3 ± 5.0 kJ mol^−1^ were derived for C_2_F_4_, C_2_Cl_4_ and C_2_Br_4_, while corresponding values of 0.6 ± 6.3, 228.1 ± 2.1 and 319.6 ± 5.4 kJ mol^−1^ were estimated for C_2_F_2_, C_2_Cl_2_ and C_2_Br_2_, respectively.

## 1. Introduction

Halogenated hydrocarbon radicals are important constituents of combustion and incineration processes [1–3]. For example, the trichloroethenyl radical (C_2_Cl_3_), as other chlorinated compounds, is involved in significant and sensitive chemical reactions during the pyrolysis of chlorine-rich unsaturated hydrocarbons. In fact, in an investigation of the kinetics and mechanism of the thermal decomposition of hexachlorocyclopentadiene from the combustion of cyclodiene pesticides, the formation of C_2_Cl_3_ radicals and dichloroethyne (C_2_Cl_2_) as fission products of *trans*-C_4_Cl_5_ radicals was suggested [4]. C_2_Cl_3_ radicals were also included in the mechanism of combustion of C_2_HCl_3_ [1] and in the pyrolysis of tetrachloroethene (C_2_Cl_4_) [2]. On the other hand, a number of both experimental and theoretical studies dealt with the reactions of C_2_Cl_3_ radicals with O_2_ [5–8], with NO_2_ [9,10] and with Cl_2_ [3], as well as with its thermal decomposition [11]. That is, in several systems of combustion and pyrolysis of unsaturated chlorinated hydrocarbons, C_2_Cl_3_ radicals have been considered as one of the most important and sensitive species, as well as the related C_2_Cl_2_ and C_2_Cl_4_ closed-shell species [12–14].

The reactions with O_2_ and NO_2_ of the analogous C_2_F_3_ radicals were also examined [15], and a vibrational characterization of the C_2_F_3_ by Fourier-transform infrared (FTIR) spectroscopy in an argon matrix was performed [16,17]. Also, this radical was proposed as a product in one of the pathways of the thermal dissociation of hexafluoropropene (C_3_F_6_) [18].

Regarding the analogous brominated species, from a time-resolved X-ray diffraction investigation, it is proposed that C_2_Br_4_ is a final stable product of photolysis of CBr_4_ in methanol [19]. However, thermochemical information on brominated hydrocarbons and their related radicals is scarce despite their role in fire suppression, as well as their potential to form a variety of environmental pollutants. As far as we know, there are theoretical estimations of the enthalpies of formation of C_2_Br_2_, C_2_Br_3_ and C_2_Br_4_ but without associated errors [20–22].

Kinetic modelling is of great importance for a better understanding of chemical reaction mechanisms. However, the success of such modelling is currently limited by the lack of information on molecular and basic thermochemical properties of the involved halogenated species. Therefore, in this work, a comparative study of structural, vibrational and thermochemical characterization of the C_2_X_*n*_, with X = F, Cl and Br and *n* = 2–4, from density functional theory (DFT) and accurate composite quantum-chemical methods was performed. Also, a detailed time-dependent DFT (TD-DFT) analysis of the UV and visible spectra of the studied species was carried out. Thus, accurate molecular and thermodynamic parameters derived here can be used for future kinetic investigations.

## 2. Computational methods

In this investigation, DFT was used, particularly with the formulations B3LYP [23], X3LYP [24], BMK [25], M06-2X [26], MN15 [27] and M08-HX [28], as implemented in the Gaussian 16 program package [29]. The large 6–311++G(3df,3pd), or 6–311+G(3df) when no H atoms are present in the molecule, and aug-cc-pVTZ basis sets were employed in all cases. Additionally, the *ab initio* composite models G3B3 [30] and G4 [31] were used, combined with the DFT optimizations: G3B3//DFT/6–311++G(3df,3pd), G3B3//DFT/aug-cc-pVTZ, G4//DFT/6–311++G(3df,3pd) and G4//DFT/aug-cc-pVTZ. Geometry optimizations were carried out without symmetry constraints using analytical gradient methods for all the studied species. Harmonic vibrational frequencies were obtained through analytical second derivative methods at the calculated equilibrium structures.

The choice of the DFT methods employed here lies in fact that most of them have been specially designed for thermochemical and kinetic studies. Also, the B3LYP performance in the treatment of the investigated group of molecules was evaluated, because it is employed in the composite methods G3B3 and G4.

The TD-DFT calculations were used to characterize electronically the excited states of the studied species. It is widely recognized that the TD-DFT gives a well-balanced description of open-shell systems, such as excited states of radicals [32]. However, to determine the most suitable DFT models to describe the absorption spectra of this group of molecules, the aforementioned formulations were employed and tested against experimental information.

## 3. Experimental details

The infrared (IR) spectrum of pure liquid C_2_Cl_4_ was recorded at room temperature with a spectral resolution of 2.0 cm^−1^ using a Nicolet 8700 FTIR spectrophotometer in attenuated total reflectance (ATR) mode, employing a ZnSe crystal. To enhance the signal-to-noise ratio, 32 scans were averaged. Subsequently, the obtained spectrum was normalized using the OMNIC 8.0 spectroscopy software prior to analysis and comparison. The chemical C_2_Cl_4_, from Sigma-Aldrich with a purity of 99.5%, was used in the experiments.

## 4. Results and discussion

### 4.1. Molecular structures and harmonic vibrational frequencies

We have no knowledge of the existence of experimental information for geometrical parameters and/or harmonic vibrational frequencies for the C_2_F_3_, C_2_Cl_3_ and C_2_Br_3_ radicals. Therefore, we first theoretically investigated the related molecules C_2_X_4_ and C_2_X_2_, with X = F, Cl and Br, for which experimental information is available [33–36], and then decided the most suitable levels of theory to estimate the properties of C_2_X_3_.

The geometrical parameters and harmonic vibrational frequencies for C_2_X_4_ and C_2_X_2_ were obtained using the B3LYP, X3LYP, BMK, M06-2X, MN15 and M08-HX functionals, combined with the 6–311+G(3df) and aug-cc-pVTZ basis sets. Particularly, their structural parameters are presented along with the experimental available information in table 1.

**Table 1.** Structural parameters calculated at different levels of theory for C_2_X_4_ and C_2_X_2_ with X = F, Cl and Br (bond lengths in Å and angles in degrees). (DFT calculations were combined with the 6–311+G(3df) and aug-cc-pVTZ (in brackets) basis sets. Experimental information is also included for C_2_F_4_ and C_2_Cl_4_ from [33], for C_2_Br_4_ from [34], for C_2_F_2_ and C_2_Cl_2_ from [35] and for C_2_Br_2_ from [36]. MAD, mean absolute deviation.)

parameters	exp.	B3LYP	X3LYP	BMK	M06-2X	MN15	M08-HX
**C_2_F_4_**							
r(C = C)	1.311	1.321 (1.321)	1.320 (1.320)	1.321 (1.321)	1.316 (1.316)	1.319 (1.319)	1.316 (1.316)
r(C–F)_av_	1.319	1.316 (1.319)	1.315 (1.318)	1.305 (1.307)	1.308 (1.311)	1.309 (1.311)	1.312 (1.316)
∠(C = C–F)_av_	123.8	123.4 (123.4)	123.4 (123.4)	123.4 (123.4)	123.3 (123.4)	123.3 (123.3)	123.3 (123.4)
∠(F–C–F)	112.4	113.2 (113.1)	113.2 (113.1)	113.3 (113.3)	113.3 (113.3)	113.3 (113.3)	113.3 (113.3)
MAD in bond lengths		0.007 (0.005)	0.007 (0.005)	0.012 (0.011)	0.008 (0.007)	0.009 (0.008)	0.006 (0.004)
MAD in bond angles		0.60 (0.55)	0.60 (0.55)	0.65 (0.65)	0.70 (0.65)	0.80 (0.80)	0.80 (0.75)
**C_2_F_2_**							
r(C≡C)	1.187	1.183 (1.183)	1.182 (1.182)	1.181 (1.181)	1.179 (1.180)	1.182 (1.182)	1.179 (1.179)
r(C–F)_av_	1.283	1.283 (1.285)	1.282 (1.284)	1.274 (1.276)	1.279 (1.282)	1.279 (1.281)	1.281 (1.285)
∠(C≡C–F)_av_		180.0 (180.0)	180.0 (180.0)	180.0 (180.0)	180.0 (180.0)	180.0 (180.0)	180.0 (180.0)
MAD in bond lengths		0.002 (0.003)	0.003 (0.003)	0.008 (0.007)	0.006 (0.004)	0.005 (0.004)	0.005 (0.005)
**C_2_Cl_4_**							
r(C = C)	1.354	1.339 (1.338)	1.338 (1.337)	1.338 (1.337)	1.334 (1.333)	1.335 (1.334)	1.333 (1.332)
r(C–Cl)_av_	1.718	1.719 (1.725)	1.717 (1.724)	1.728 (1.734)	1.708 (1.713)	1.670 (1.703)	1.707 (1.712)
∠(C = C–Cl)_av_	122.15	122.5 (122.5)	122.5 (122.5)	122.4 (122.4)	122.3 (122.4)	122.3 (122.3)	122.2 (122.2)
∠(Cl–C–Cl)	115.7	115.0 (115.0)	115.1 (115.1)	115.1 (115.1)	115.3 (115.3)	115.5 (115.5)	115.5 (115.6)
MAD in bond lengths		0.008 (0.012)	0.009 (0.012)	0.013 (0.017)	0.015 (0.013)	0.034 (0.018)	0.016 (0.014)
MAD in bond angles		0.53 (0.53)	0.45 (0.48)	0.43 (0.43)	0.28 (0.33)	0.18 (0.18)	0.13 (0.08)
**C_2_Cl_2_**							
r(C≡C)	1.246	1.198 (1.199)	1.197 (1.198)	1.195 (1.196)	1.194 (1.195)	1.196 (1.196)	1.194 (1.194)
r(C–Cl)_av_	1.612	1.635 (1.642)	1.635 (1.641)	1.652 (1.659)	1.634 (1.639)	1.629 (1.633)	1.633 (1.638)
∠(C≡C–Cl)_av_	180	179.9 (180.0)	180.0 (180.0)	180.0 (180.0)	180.0 (180.0)	180.0 (180.0)	180.0 (180.0)
MAD in bond lengths		0.036 (0.039)	0.036 (0.039)	0.046 (0.049)	0.037 (0.039)	0.034 (0.036)	0.037 (0.039)
MAD in bond angles		0.10 (0.00)	0.00 (0.00)	0.00 (0.00)	0.00 (0.00)	0.00 (0.00)	0.00 (0.00)
**C_2_Br_4_**							
r(C = C)	1.362	1.336 (1.336)	1.335 (1.335)	1.339 (1.338)	1.333 (1.332)	1.334 (1.333)	1.333 (1.332)
r(C–Br)_av_	1.881	1.891 (1.892)	1.890 (1.890)	1.882 (1.885)	1.875 (1.877)	1.867 (1.865)	1.873 (1.875)
∠(C = C–Br)_av_		123.0 (123.0)	123.0 (122.9)	122.9 (122.9)	122.8 (122.8)	122.7 (122.7)	122.6 (122.6)
∠(Br–C–Br)	115.2	114.1 (114.1)	114.1 (114.1)	114.2 (114.2)	114.3 (114.3)	114.5 (114.7)	114.9 (114.9)
MAD in bond lengths		0.018 (0.019)	0.018 (0.018)	0.012 (0.014)	0.018 (0.017)	0.021 (0.023)	0.019 (0.018)
MAD in bond angles		1.10 (1.10)	1.10 (1.10)	1.00 (1.00)	0.90 (0.90)	0.70 (0.50)	0.50 (0.50)
**C_2_Br_2_**							
r(C≡C)	1.205	1.201 (1.201)	1.200 (1.201)	1.199 (1.200)	1.197 (1.198)	1.200 (1.200)	1.197 (1.197)
r(C–Br)_av_	1.79	1.794 (1.795)	1.793 (1.794)	1.794 (1.798)	1.788 (1.790)	1.784 (1.783)	1.785 (1.787)
∠(C≡C–Br)_av_		180.0 (180.0)	180.0 (180.0)	180.0 (180.0)	180.0 (180.0)	180.0 (180.0)	180.0 (180.0)
MAD in bond lengths		0.004 (0.005)	0.004 (0.004)	0.005 (0.007)	0.005 (0.004)	0.006 (0.006)	0.007 (0.006)

**Figure 1.**
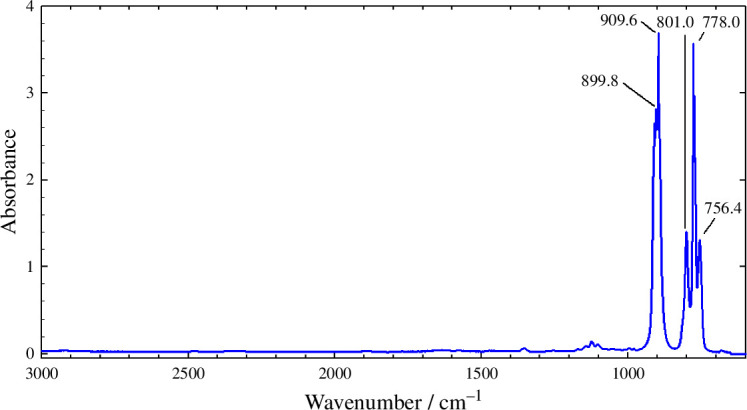
Experimental IR spectrum of pure liquid C_2_Cl_4_ at room temperature.

As it is expected, the C_2_X_4_ molecules lie on a plane, while the C_2_X_2_ present linear structure. Another known characteristic that can be observed is the shortening of the CC bond lengths when passing from brominated to chlorinated substituents, with the smallest bond lengths observed for the fluorinated compounds [37]. This shortening is similar in C_2_X_4_ and in C_2_X_2_ species. For example, values of 1.339 Å (C_2_Br_4_), 1.338 Å (C_2_Cl_4_), 1.321 Å (C_2_F_4_), 1.199 Å (C_2_Br_2_), 1.195 Å (C_2_Cl_2_), and 1.181 Å (C_2_F_2_), were derived at the BMK/6–311+G(3df) level. All the functionals employed here give very good results, but the B3LYP method shows the smallest mean absolute deviations (MAD) from the experimental values with both used basis sets. In some cases, the calculations with the Pople basis set give smaller MAD values than those with the Dunning basis set, while in others, this is reversed. On the other hand, the calculated harmonic vibrational frequencies are listed in tables 2 and 3. It should be noted that owing to the high symmetry of these species, several vibrational modes are not active in the IR. Therefore, experimental vibrational frequencies included in these tables were obtained from IR spectroscopy or from Raman spectroscopy when the modes are IR inactive [38–42]. A low dispersion is observed among the calculated values, being again the B3LYP/6–311+G(3df) level the method with the smallest MAD values, although all the functionals give acceptable results. In addition, the obtained intensities show good agreement with the available experimental information. The C_2_X_4_ compounds possess twelve vibrational modes, nine of which are movements in the plane of the molecules (five stretching, three bending and one rocking), while three are out of it (two involving C out-of-plane motions and one torsional mode). In the case of C_2_X_2_ species, they present seven vibrational modes, with three entailing movements along the molecular axis (stretching modes) and the remaining four are out of this axis. Moreover, as it is expected, a decrease in the C_2_X_4_ vibrational frequencies is observed when increasing halogen mass. For the C_2_X_2_ family, the behaviour is mostly the same, except for certain vibrational modes associated with XCC movements where the frequencies are higher for C_2_Cl_2_. Additionally, figure 1 shows the IR spectrum of pure liquid C_2_Cl_4_, determined experimentally in our laboratory, with a Nicolet 8700 FTIR spectrophotometer in ATR mode. It is possible to highlight the good agreement observed between the vibrational frequencies calculated in this research and the experimental available information (table 2). As can be seen from figure 1, two high-intensity peaks corresponding to the symmetrical (778.0 cm^−1^) and asymmetrical stretching modes of CCl_2_ (909.6 cm^−1^) are observed.

**Table 2.** Harmonic vibrational frequencies (cm^−1^) and approximate assignments calculated at different levels of theory for C_2_F_4_, C_2_Cl_4_ and C_2_Br_4_. (DFT calculations were combined with the 6-311+G(3df) and aug-cc-pVTZ (in brackets) basis sets. Experimental information is also included for C_2_F_4_, C_2_Cl_4_ and C_2_Cl_4_ [38]. S, strong; M, medium; W, weak. MAD, mean absolute deviation.)

approximate assignments	exp.	B3LYP	X3LYP	BMK	M06-2X	MN15	M08-HX
**C_2_F_4_**							
stretching C = C	1872^a^	1906 (1905)	1914 (1913)	1939 (1927)	1960 (1963)	1962 (1957)	1956 (1959)
stretching asym. CF_2_	1340^a^	1329 (1323)	1336 (1330)	1426 (1420)	1403 (1396)	1399 (1394)	1369 (1361)
stretching asym. CF_2_	1337 S	1325 (1320)	1331 (1328)	1413 (1409)	1399 (1394)	1397 (1392)	1362 (1357)
stretching sym. CF_2_	1186 S	1186 (1182)	1192 (1187)	1245 (1242)	1230 (1229)	1231 (1227)	1207 (1202)
stretching sym. CF_2_	778^a^	796 (794)	799 (797)	829 (827)	824 (821)	823 (820)	811 (807)
C out of plane	558 S	559 (554)	564 (559)	573 (571)	586 (582)	603 (590)	602 (597)
bending CCF	551^a^	556 (553)	558 (555)	573 (571)	567 (565)	560 (557)	565 (562)
bending CF_2_	508^a^	555 (553)	557 (555)	571 (564)	564 (564)	558 (554)	559 (556)
C out of plane	406 S	417 (415)	418 (417)	420 (411)	427 (428)	431 (427)	433 (430)
bending CF_2_	394^a^	400 (398)	400 (399)	409 (407)	403 (404)	398 (397)	399 (398)
rocking CF_2_	218 S	212 (210)	211 (210)	219 (215)	209 (214)	205 (202)	209 (207)
torsion	190^a^	200 (199)	201 (200)	207 (207)	205 (206)	201 (201)	200 (203)
MAD		13 (14)	15 (14)	41 (37)	38 (36)	38 (34)	29 (27)
**C_2_Cl_4_**							
stretching C = C	1571^a^	1612 (1615)	1618 (1622)	1641 (1634)	1657 (1667)	1676 (1679)	1676 (1679)
stretching asym. CCl_2_	1000^a^	966 (956)	972 (962)	1030 (1032)	1014 (999)	1034 (1008)	1051 (1037)
stretching asym. CCl_2_	908 S	884 (875)	890 (880)	950 (955)	937 (928)	957 (957)	967 (962)
stretching sym. CCl_2_	777 S	776 (767)	779 (771)	808 (802)	806 (801)	813 (808)	816 (808)
C out of plane	512^a^	564 (543)	567 (546)	561 (533)	569 (548)	591 (559)	579 (555)
stretching sym. CCl_2_	447^a^	450 (444)	451 (446)	466 (465)	467 (462)	470 (468)	473 (469)
bending CCCl	347^a^	348 (344)	349 (346)	355 (351)	357 (353)	356 (348)	359 (354)
bending CCl_2_	310 W	317 (311)	318 (312)	313 (306)	319 (312)	319 (312)	317 (309)
C out of plane	288 M	296 (291)	296 (292)	281 (274)	276 (274)	294 (290)	285 (282)
bending CCl_2_	237^a^	240 (236)	241 (237)	243 (237)	243 (239)	240 (235)	239 (234)
rocking CCl_2_	176 S	179 (177)	180 (177)	189 (184)	184 (181)	180 (176)	179 (176)
torsion	110^a^	99 (98)	99 (99)	96 (95)	94 (94)	103 (103)	99 (99)
MAD		16 (16)	16 (15)	24 (21)	25 (20)	30 (23)	32 (24)
**C_2_Br_4_**							
stretching C = C	1535^a^	1577 (1578)	1583 (1585)	1599 (1589)	1621 (1626)	1630 (1632)	1631 (1634)
stretching asym. CBr_2_	880^a^	867 (869)	872 (874)	928 (928)	930 (930)	909 (909)	906 (900)
stretching asym. CBr_2_	766 S	750 (751)	755 (756)	808 (811)	815 (817)	803(806)	795 (791)
stretching sym. CBr_2_	635 S	633 (632)	636 (635)	682 (677)	678 (674)	660 (660)	662 (660)
C out of plane	464^a^	497 (497)	500 (499)	511 (506)	533 (531)	521 (521)	518 (514)
stretching sym. CBr_2_	265^a^	268 (268)	268 (269)	281 (281)	284 (285)	279 (280)	281 (280)
C out of plane	245 S	250 (249)	250 (249)	249 (248)	284 (283)	251 (252)	248 (245)
bending CCBr	208^a^	212 (212)	213 (213)	222 (221)	222 (221)	217 (217)	221 (221)
bending CBr_2_	188 M	190 (189)	190 (190)	197 (194)	202 (201)	191 (189)	196 (193)
bending CBr_2_	144^a^	146 (145)	146 (145)	149 (147)	154 (154)	147 (146)	152 (151)
rocking CBr_2_	119 M	117 (117)	118 (117)	121 (119)	127 (127)	120 (119)	124 (124)
torsion	66^a^	55 (55)	56 (56)	57 (56)	67 (68)	59 (60)	57 (54)
MAD		11 (11)	11 (11)	26 (24)	34 (34)	24 (24)	25 (23)

^a^ IR inactive.

**Table 3.** Harmonic vibrational frequencies (cm^−1^) and approximate assignments calculated at different levels of theory for C_2_F_2_, C_2_Cl_2_, and C_2_Br_2_. (DFT calculations were combined with the 6–311+G(3df) and aug-cc-pVTZ (in brackets) basis sets. Experimental information is also included for C_2_F_2_ [39,40], and for C_2_Cl_2_ and C_2_Br_2_ [41]. VS very strong; S, strong; W, weak. MAD, mean absolute deviation.)

approximate assignments	exp.	B3LYP	X3LYP	BMK	M06−2X	MN15	M08-HX
**C_2_F_2_**							
stretching CC	2436^a^	2550 (2550)	2557 (2557)	2557 (2557)	2613 (2616)	2590 (2591)	2596 (2602)
stretching asym. CF	1350 VS	1369 (1365)	1374 (1370)	1426 (1424)	1412 (1407)	1412 (1409)	1380 (1373)
stretching sym. CF	787^a^	800 (797)	802 (800)	824 (824)	822 (819)	819 (818)	807 (804)
bending FCC		358 (322)	365 (330)	380 (343)	428 (402)	437 (414)	429 (399)
bending FCC		358 (322)	365 (330)	380 (343)	428 (402)	437 (414)	429 (399)
bending FCC	270 W	287 (281)	288 (283)	271 (256)	304 (301)	296 (293)	298 (293)
bending FCC	268 W	287 (281)	288 (283)	271 (256)	304 (301)	296 (293)	298 (293)
MAD		36 (33)	40 (36)	48 (52)	69 (67)	60 (59)	54 (51)
**C_2_Cl_2_**							
stretching CC	2234 ± 10^a^	2317 (2318)	2323 (2325)	2318 (2323)	2386 (2388)	2362 (2366)	2377 (2379)
stretching asym. CCl	988 ± 2 VS	1003 (992)	1006 (995)	1005 (994)	1030 (1026)	1030 (1026)	1027 (1018)
stretching sym. CCl	477 ± 10^a^	481 (476)	483 (478)	480 (475)	494 (491)	494 (491)	492 (487)
bending ClCC		426 (371)	429 (375)	432 (365)	472 (413)	480 (438)	483 (409)
bending ClCC	333 ± 10^a^	426 (371)	429 (375)	432 (365)	472 (413)	480 (438)	483 (409)
bending ClCC		186 (179)	187 (180)	166 (153)	184 (181)	194 (191)	181 (174)
bending ClCC	172 ± 2 S	186 (179)	187 (180)	166 (153)	184 (181)	194 (191)	181 (174)
MAD		42 (27)	45 (30)	42 (30)	72 (59)	71 (62)	71 (53)
**C_2_Br_2_**							
stretching CC	2185^a^	2269 (2270)	2276 (2277)	2275 (2279)	2338 (2336)	2310 (2312)	2332 (2335)
stretching asym. CBr	832 VS	839 (834)	842 (837)	871 (866)	865 (860)	867 (870)	875 (874)
bending BrCC		352 (348)	356 (351)	366 (359)	408 (402)	395 (404)	398 (388)
bending BrCC	311^a^	352 (348)	356 (351)	366 (359)	408 (402)	395 (404)	398 (388)
stretching sym. CBr	267^a^	290 (289)	291 (290)	299 (298)	299 (297)	299 (300)	302 (302)
bending BrCC		142 (144)	142 (144)	144 (145)	174 (180)	144 (146)	155 (153)
bending BrCC	137 S	142 (144)	142 (144)	144 (145)	174 (180)	144 (146)	155 (153)
MAD		32 (31)	35 (34)	45 (43)	70 (69)	57 (60)	66 (64)

^a^ IR inactive.

**Figure 2.**
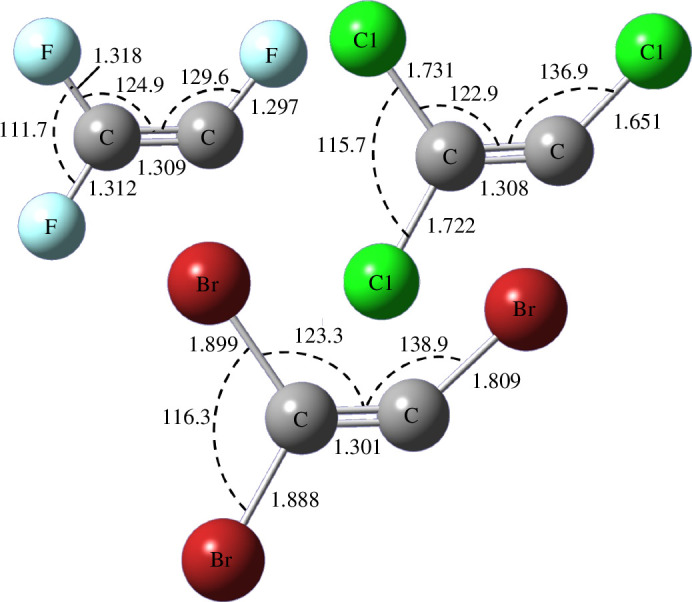
Geometrical structures calculated for C_2_F_3_, C_2_Cl_3_ and C_2_Br_3_ with the average parameters from table 4 (interatomic distances in Å and angles in degrees).

The previous evaluation shows no important differences between the employed methods, therefore, all of them were used to estimate geometrical parameters and harmonic vibrational frequencies for the C_2_X_3_ radicals (with X = F, Cl and Br). Table 4 lists the computed structural parameters, where the aforementioned is also evidenced, and figure 2 presents the molecular geometries for the three radicals with the average values of bond lengths and bond angles. As can be seen, these molecules are planar, with dihedral angles X-C=CX of 0°. The C=C bond lengths are shorter compared to C_2_X_4_, while the C-X distances are similar to the C_2_X_4_, except when C is attached to a single halogen, in which case they are shorter. In addition, some differences are observed in the X-C=C angles of the C_2_X_3_ radicals, expected as a consequence of its asymmetry.

**Table 4.** Structural parameters calculated at different levels of theory for C_2_X_3_ with X = F, Cl and Br (bond lengths in Å and angles in degrees). (DFT calculations were combined with the 6–311+G(3df) and aug-cc-pVTZ (in brackets) basis sets. The average of the calculated values are also presented.)

parameters	B3LYP	X3LYP	BMK	M06-2X	MN15	M08-HX	average
**C_2_F_3_**							
r(C = C)	1.311 (1.311)	1.310 (1.310)	1.313 (1.314)	1.308 (1.308)	1.309 (1.309)	1.308 (1.307)	1.309 (1.310)
r(C–F)	1.324 (1.327)	1.323 (1.326)	1.311 (1.313)	1.314 (1.317)	1.315 (1.317)	1.319 (1.324)	1.318 (1.321)
r(C–F)	1.317 (1.320)	1.316 (1.319)	1.305 (1.307)	1.309 (1.312)	1.310 (1.312)	1.313 (1.317)	1.312 (1.315)
r(C–F)	1.302 (1.307)	1.300 (1.303)	1.291 (1.293)	1.297 (1.300)	1.295 (1.297)	1.298 (1.302)	1.297 (1.300)
∠(F–C = C)	125.0 (125.0)	124.9 (125.0)	124.8 (124.8)	124.8 (124.9)	124.9 (124.9)	124.8 (124.9)	124.9 (124.9)
∠(C = C–F)	130.0 (129.9)	129.9 (129.8)	129.1 (129.0)	128.7 (128.6)	130.0 (129.9)	129.7 (129.7)	129.6 (129.5)
∠(F–C–F)	111.7 (111.7)	111.7 (111.7)	111.8 (111.8)	111.8 (111.7)	111.7 (111.7)	111.7 (111.7)	111.7 (111.7)
**C_2_Cl_3_**							
r(C = C)	1.308 (1.307)	1.307 (1.306)	1.309 (1.308)	1.307 (1.306)	1.309 (1.306)	1.306 (1.305)	1.308 (1.307)
r(C–Cl)	1.739 (1.747)	1.738 (1.745)	1.743 (1.749)	1.723 (1.729)	1.718 (1.721)	1.723 (1.729)	1.731 (1.737)
r(C–Cl)	1.729 (1.735)	1.727 (1.734)	1.736 (1.742)	1.716 (1.722)	1.709 (1.713)	1.715 (1.720)	1.722 (1.728)
r(C–Cl)	1.650 (1.658)	1.650 (1.658)	1.671 (1.680)	1.649 (1.656)	1.639 (1.645)	1.645 (1.652)	1.651 (1.658)
∠(Cl–C = C)	123.2 (123.3)	123.2 (123.3)	123.1 (123.1)	122.8 (122.9)	122.6 (122.7)	122.5 (122.5)	122.9 (123.0)
∠(C = C–Cl)	138.2 (138.0)	138.1 (137.8)	135.8 (135.4)	135.9 (135.5)	136.7 (136.4)	136.4 (136.1)	136.9 (136.5)
∠(Cl–C–Cl)	115.6 (115.5)	115.6 (115.5)	115.6 (115.6)	115.9 (115.9)	115.6 (115.6)	115.9 (115.8)	115.7 (115.7)
**C_2_Br_3_**							
r(C = C)	1.300 (1.300)	1.300 (1.299)	1.304 (1.304)	1.301 (1.301)	1.301 (1.301)	1.301 (1.300)	1.301 (1.301)
r(C–Br)	1.915 (1.916)	1.912 (1.914)	1.898 (1.901)	1.890 (1.892)	1.887 (1.885)	1.890 (1.893)	1.899 (1.900)
r(C–Br)	1.901 (1.902)	1.899 (1.900)	1.891 (1.894)	1.882 (1.884)	1.876 (1.875)	1.881 (1.883)	1.888 (1.890)
r(C–Br)	1.814 (1.815)	1.813 (1.814)	1.815 (1.819)	1.809 (1.811)	1.796 (1.796)	1.805 (1.806)	1.809 (1.810)
∠(Br–C = C)	123.7 (123.8)	123.6 (123.7)	123.5 (123.6)	123.2 (123.2)	122.9 (123.0)	122.8 (122.7)	123.3 (123.3)
∠(C = C–Br)	140.3 (140.3)	140.2 (140.2)	138.6 (138.4)	137.8 (137.7)	138.8 (138.5)	137.7 (137.7)	138.9 (138.8)
∠(Br–C–Br)	116.1 (116.0)	116.1 (116.1)	116.4 (116.4)	116.7 (116.7)	116.1 (116.1)	116.6 (116.6)	116.3 (116.3)

Tables 5 and 6 show the harmonic vibrational frequencies and IR intensities for the C_2_X_3_ radicals (with X = F, Cl and Br), computed employing the aforementioned functionals. Similar results are observed for both used basis sets. The C_2_X_3_ radicals exhibit nine vibrational modes, of which seven are movements in the plane of the molecules (four stretching and three bending) and two out of this plane. Furthermore, as it is expected, a decrease in the vibrational frequencies when the halogen mass increases is observed.

**Table 5.** Harmonic vibrational frequencies (cm^−1^), approximate assignments and infrared intensities (in brackets, in km mol^−1^) calculated at different levels of theory for C_2_X_3_ radicals (with X = F, Cl and Br) coupled with the 6–311+G(3df) basis set. (The average of the calculated values are also presented.)

approximate assignments	B3LYP	X3LYP	BMK	M06-2X	MN15	M08-HX	average
**C_2_F_3_**							
stretching C = C	1845 (44)	1852 (44)	1867 (49)	1889 (50)	1904 (47)	1888 (47)	1874 (47)
stretching asym. CF_2_	1279 (325)	1286 (322)	1367 (296)	1349 (279)	1347 (300)	1315 (288)	1324 (302)
stretching CF	1226 (266)	1232 (270)	1301 (324)	1281 (302)	1286 (311)	1254 (297)	1263 (295)
stretching sym. CF_2_	919 (26)	923 (26)	959 (23)	956 (27)	948 (24)	938 (27)	940 (26)
bending FCC	620 (1)	622 (1)	638 (2)	632 (2)	622 (2)	626 (2)	627 (2)
C out of plane	503 (7)	507 (7)	518 (9)	542 (7)	547 (7)	542 (7)	527 (7)
bending CF_2_	484 (1)	486 (2)	498 (2)	493 (2)	486 (2)	489 (2)	489 (2)
C out of plane	259 (0.8)	262 (0.7)	263 (1)	301 (0.01)	292 (0.003)	292 (0.03)	278 (0.4)
bending FCC	214 (4)	215 (4)	217 (4)	215 (4)	202 (4)	204 (4)	211 (4)
**C_2_Cl_3_**							
stretching C = C	1697 (5)	1704 (5)	1697 (5)	1722 (6)	1747 (6)	1744 (5)	1719 (5)
stretching asym. CCl_2_	859 (149)	863 (149)	939 (134)	896 (141)	924 (147)	934 (128)	903 (141)
stretching CCl	821 (134)	825 (133)	870 (120)	860 (122)	878 (124)	874 (110)	855 (124)
stretching sym. CCl_2_	582 (3)	585 (3)	616 (0.7)	608 (2)	612 (2)	614 (1)	603 (2)
C out of plane	464 (0.03)	466 (0.03)	461 (0.01)	469 (0.01)	489 (0.1)	481 (0.1)	472 (0.05)
bending ClCC	391 (1)	392 (1)	397 (0.8)	396 (0.9)	398 (1)	401 (0.7)	396 (0.9)
bending CCl_2_	263 (0.01)	264 (1)	262 (0.1)	264 (0.1)	264 (0.1)	263 (0.1)	263 (0.2)
C out of plane	194 (0.9)	196 (0.3)	195 (0.9)	183 (0.6)	202 (0.4)	190 (0.6)	193 (0.6)
bending ClCC	129 (1)	130 (0.3)	136 (1)	128 (1)	130 (1)	126 (1)	130 (0.9)
**C_2_Br_3_**							
stretching C = C	1683 (4)	1688 (4)	1674 (4)	1717 (3)	1724 (4)	1729 (4)	1703 (4)
stretching asym. CBr_2_	722 (115)	728 (115)	796 (112)	784 (107)	778 (103)	778 (125)	764 (113)
stretching CBr	675 (117)	680 (114)	721 (99)	722 (96)	721 (95)	725 (100)	707 (104)
stretching sym. CBr_2_	462 (2)	465 (2)	491 (0.8)	494 (0.8)	483 (0.6)	492 (0.8)	481 (1)
C out of plane	413 (0.1)	416 (0.1)	424 (0.1)	438 (0.1)	439 (0.1)	438 (0.1)	428 (0.1)
bending BrCC	240 (0.9)	242 (0.9)	251 (0.7)	251 (0.7)	247 (0.9)	251 (0.7)	247 (0.8)
C out of plane	160 (1)	161 (1)	167 (2)	178 (1)	166 (1)	164 (1)	166 (1)
bending CBr_2_	153 (0.003)	153 (0.002)	157 (0.001)	159 (0.01)	154 (0.01)	159 (0.002)	156 (0.005)
bending BrCC	80 (0.7)	80 (0.7)	84 (0.6)	87 (0.6)	81 (0.6)	81 (0.7)	82 (0.7)

**Table 6.** Harmonic vibrational frequencies (in cm^−1^), approximate assignments and infrared intensities (in brackets, in km mol^−1^) calculated at different levels of theory for C_2_X_3_ radicals (with X = F, Cl and Br) coupled with the aug-cc-pVTZ basis set. (The average of the calculated values are also presented.)

approximate assignments	B3LYP	X3LYP	BMK	M06-2X	MN15	M08-HX	average
**C_2_F_3_**							
stretching C = C	1843 (43)	1850 (44)	1858 (49)	1890 (51)	1898 (48)	1891 (48)	1872 (47)
stretching asym. CF_2_	1274 (326)	1281 (323)	1365 (301)	1344 (278)	1345 (302)	1310 (293)	1320 (304)
stretching CF	1222 (262)	1228 (267)	1300 (325)	1273 (303)	1283 (308)	1247 (291)	1259 (293)
stretching sym. CF_2_	916 (26)	921 (26)	958 (24)	952 (27)	945 (23)	934 (27)	938 (26)
bending FCC	617 (1)	619 (1)	636 (2)	629 (2)	620 (2)	623 (2)	624 (2)
C out of plane	500 (7)	505 (7)	511 (8)	541 (7)	540 (7)	537 (7)	522 (7)
bending CF_2_	483 (1)	484 (2)	497 (2)	493 (2)	484 (2)	487 (2)	488 (2)
C out of plane	255 (0.9)	258 (0.8)	257 (1)	293 (0.01)	288 (0.02)	289 (0.02)	273 (0.5)
bending FCC	212 (4)	213 (4)	216 (4)	215 (4)	203 (4)	205 (4)	211 (4)
**C_2_Cl_3_**							
stretching C = C	1699 (5)	1704 (5)	1691 (0.8)	1726 (6)	1744 (6)	1746 (5)	1718 (5)
stretching asym. CCl_2_	848 (152)	853 (152)	936 (138)	886 (144)	920 (148)	921 (130)	894 (144)
stretching CCl	810 (136)	815 (135)	865 (121)	850 (122)	872 (125)	862 (111)	846 (125)
stretching sym. CCl_2_	576 (3)	579 (3)	613 (0.8)	603 (2)	608 (2)	608 (1)	598 (2)
C out of plane	455 (0.004)	458 (0.004)	451 (0.001)	462 (0.0002)	478 (0.03)	471 (0.02)	463 (2)
bending ClCC	386 (1)	387 (1)	392 (0.7)	391 (0.8)	393 (1)	394 (0.7)	391 (0.9)
bending CCl_2_	259 (0.02)	260 (0.03)	258 (0.1)	258 (0.08)	259 (0.1)	257 (0.1)	259 (0.07)
C out of plane	193 (0.9)	194 (0.8)	189 (0.8)	182 (0.5)	201 (0.4)	189 (0.6)	191 (0.7)
bending ClCC	128 (1)	128 (1)	134 (1)	124 (1)	126 (1)	123 (1)	127 (1)
**C_2_Br_3_**							
stretching C = C	1686 (4)	1691 (4)	1670 (5)	1719 (4)	1723 (4)	1732 (4)	1704 (4)
stretching asym. CBr_2_	719 (114)	725 (114)	793 (111)	777 (105)	777 (101)	775 (123)	761 (111)
stretching CBr	673 (118)	677 (115)	719 (99)	718 (93)	722 (95)	722 (100)	705 (103)
stretching sym. CBr_2_	460 (2)	463 (2)	490 (0.8)	492 (0.7)	482 (0.6)	490 (0.8)	480 (1)
C out of plane	412 (0.1)	415 (0.1)	421 (0.1)	435 (0.1)	438 (0.05)	438 (0.09)	427 (0.09)
bending BrCC	240 (0.9)	241 (0.9)	250 (0.7)	249 (0.7)	246 (0.9)	250 (0.7)	246 (0.8)
C out of plane	160 (1)	161 (1)	166 (2)	181 (1)	167 (1)	161 (1)	166 (1)
bending CBr_2_	153 (0.002)	153 (0.001)	156 (0.002)	158 (0.01)	153 (0.01)	158 (0.005)	155 (2)
bending BrCC	80 (0.7)	80 (0.7)	84 (0.6)	88 (0.5)	79 (0.6)	80 (0.7)	82 (0.6)

### 4.2. Ultraviolet and visible absorption spectrum

The TD-DFT calculations were used to derive the vertical electronic energies, *E*_max_, the associated wavelengths of the band maxima, λ_max_, and oscillator strengths, *f*, of the investigated species. In order to select the best models to describe the absorption spectra of this group of molecules, the results were compared against the available experimental information. The electronic supplementary material, tables S1–S6 list, list the results obtained with the B3LYP, X3LYP, BMK, M06-2X, MN15 and M08-HX functionals, combined with the 6–311+G(3df) and the aug-cc-pVTZ basis sets. As it is observed, the use of different basis sets does not significantly affect the excitation energy (or wavelength) and oscillator strength. Also, despite the different generations of exchange and correlation functionals employed, similar results were found and, thus, no approach can be definitely attributed superiority over the other. Therefore, the obtained values were averaged for each species to facilitate the comparison with available experimental and theoretical data. In this way, the TD-DFT average values (with the corresponding standard deviation) of *E*_max_ = 8.4 ± 0.2 eV (λ_max_ = 148 ± 5 nm) and *f* = 0.35 ± 0.05 were obtained for the most intense transition of C_2_F_4_. The available experimental information of C_2_F_4_ indicates an absorption band over the 185–209 nm range with increasing intensity at shorter wavelengths [43]. This observation is in reasonable agreement with the computed data listed in the electronic supplementary material, tables S1 and S2, with an absorption starting at about 6.5 eV (191 nm). Regarding C_2_Cl_4_, the TD-DFT average values of *E*_max_ = 5.9 ± 0.1 eV (λ_max_ = 210 ± 8 nm) and *f* = 0.37 ± 0.01 were derived, corresponding to the most intense absorption. These results are in good agreement with the known experimental information [44–46]. There is another calculated transition with lower intensity at 7.6 ± 0.3 eV that also agrees very well with the reported spectrum [44]. On the other hand, the values derived here for C_2_Br_4_ are *E*_max_ = 5.6 ± 0.1 eV (λ_max_ = 221 ± 10 nm) and *f* = 0.31 ± 0.04. They are in excellent agreement with the available experimental information, which depicts an absorption in the range of 216–223 nm with a band maximum at 219.5 nm (5.65 eV) [47]. It can be seen that the energy of the maximum of the most intense band decreases as the halogen changes from F, Cl to Br in C_2_X_4_. A similar trend can be observed in the electronic supplementary material, tables S3 and S4, for C_2_X_2_, although C_2_F_2_ presents an additional intense transition at lower energy. The resultant average values for *E*_max_, λ_max_ and *f* for C_2_F_2_ are 6.8 ± 0.3 eV (λ_max_ = 182 ± 16 nm) with *f* = 0.05 ± 0.01 and 9.7 ± 0.5 eV (λ_max_ = 127 ± 13 nm) with *f* = 0.04 ± 0.01. A good agreement is observed with the calculated cross sections for electron collision with difluoroacetylene reported in the study of Gupta *et al*. [48]. In the case of C_2_Cl_2_, the obtained TD-DFT average values are of 8.8 ± 0.4 eV (λ_max_ = 182 ± 14 nm) with *f* = 0.9 ± 0.4, while for C_2_Br_2_, average values of *E*_max_ = 8.0 ± 0.3 eV (λ_max_ = 155 ± 11 nm) and *f* = 1.1 ± 0.3, were derived. As far as we know, there is no experimental UV spectra information about C_2_X_2_ and C_2_X_3_ (X = F, Cl and Br) species to compare. Based on the results presented above for C_2_X_4_, the TD-DFT values derived here seem to be a reasonable approximation. The electronic supplementary material, tables S5 and S6, include all the results for C_2_F_3_, C_2_Cl_3_ and C_2_Br_3_ radicals calculated using different functionals with the 6–311+G(3df) and aug-cc-pVTZ basis sets, while tables 7 and 8 summarize the corresponding average values for the more significant electronic states of C_2_X_*n*_ (with X = F, Cl and Br and *n* = 2–4). The resulting average values for *E*_max_, λ_max_ and *f* associated with the band maxima for C_2_X_3_ are *E*_max_ = 8.1 ± 0.2 eV (λ_max_ = 153 ± 9 nm) and *f* = 0.13 ± 0.05 for C_2_F_3_; *E*_max_ = 6.37 ± 0.05 eV (λ_max_ = 195 ± 3 nm) and *f* = 0.25 ± 0.06 for C_2_Cl_3_; and *E*_max_ = 6.1 ± 0.1 eV (λ_max_ = 204 ± 9 nm) and *f* = 0.23 ± 0.06 for C_2_Br_3_. As can be seen from the tables, the energy of the maximum of the most intense band is lower when the halogen changes from F, Cl to Br. Furthermore, it is evident that absorption begins at lower energies than the band maximum. The UV absorption of C_2_F_3_, C_2_Cl_3_ and C_2_Br_3_ radicals begins, respectively, at about 6, 5 and 4 eV.

**Table 7.** Vertical excitation energies, *E*, (eV) and oscillator strengths, *f*, (in brackets) for the more significant electronic states of C_2_X_*n*_ (X = F, Cl and Br and *n* = 2–4), calculated using different functionals with the 6–311+G(3df) basis set.

species	B3LYP	X3LYP	BMK	M06-2X	MN15	M08-HX	average
C_2_F_4_	8.26 (0.3661)	8.29 (0.3638)	8.56 (0.3995)	8.47 (0.3604)	8.25 (0.4000)	8.37 (0.2557)	8.37 (0.36)
C_2_Cl_4_	5.73 (0.3551)	5.75 (0.3597)	6.01 (0.3843)	6.01 (0.3862)	5.85 (0.3836)	6.02 (0.3797)	5.90 (0.37)
C_2_Br_4_	5.41 (0.2934)	5.44 (0.3009)	5.67 (0.3465)	5.70 (0.3295)	5.53 (0.3424)	5.73 (0.2050)	5.58 (0.30)
C_2_F_2_	6.63 (0.0454)	6.60 (0.0451)	7.06 (0.0639)	6.91 (0.0468)	7.36 (0.0633)	6.51 (0.0412)	6.85 (0.05)
	9.62 (0.0479)	9.63 (0.0478)	10.12(0.0501)	9.97 (0.0481)	10.90(0.0396)	9.53 (0.0505)	9.96 (0.05)
C_2_Cl_2_	8.43 (1.0196)	8.43 (0.9978)	9.38 (1.1159)	8.82 (1.1450)	9.02 (1.7080)	8.52 (0.7023)	8.77 (1.11)
C_2_Br_2_	7.67 (0.9873)	7.68 (0.9758)	8.23 (1.3554)	8.08 (1.1468)	8.25 (1.5974)	7.71 (0.7648)	7.94 (1.14)
C_2_F_3_	8.49 (0.1361)	8.48 (0.1592)	8.14 (0.1432)	7.94 (0.1163)	8.03 (0.2049)	7.89 (0.0559)	8.16 (0.14)
C_2_Cl_3_	6.29 (0.2860)	6.31 (0.2850)	6.40 (0.1667)	6.41 (0.3100)	6.33 (0.2751)	6.40 (0.2894)	6.36 (0.27)
C_2_Br_3_	5.91 (0.2757)	5.93 (0.2804)	6.23 (0.2819)	6.24 (0.2198)	6.13 (0.1686)	6.05 (0.1209)	6.08 (0.22)

**Table 8.** Vertical excitation energies, *E*, (eV) and oscillator strengths, *f*, (in brackets) for the more significant electronic states of C_2_X_*n*_ (X = F, Cl and Br and *n* = 2–4), calculated using different functionals with the aug-cc-pVTZ basis set.

species	B3LYP	X3LYP	BMK	M06−2X	MN15	M08-HX	average
C_2_F_4_	8.24 (0.3353)	8.27 (0.3297)	8.51 (0.3792)	8.46 (0.3376)	8.23 (0.3952)	8.70 (0.2362)	8.40 (0.34)
C_2_Cl_4_	5.78 (0.3538)	5.81 (0.3592)	6.07 (0.3788)	6.04 (0.3880)	5.90 (0.3827)	6.05 (0.3830)	5.94 (0.37)
C_2_Br_4_	5.40 (0.2886)	5.43 (0.2959)	5.67 (0.3339)	5.70 (0.2839)	5.53 (0.3373)	5.70 (0.3316)	5.57 (0.31)
C_2_F_2_	6.59 (0.0438)	6.57 (0.0437)	6.97 (0.0647)	6.92 (0.0469)	7.31 (0.0642)	6.52 (0.0381)	6.81 (0.05)
	9.15 (0.0371)	9.18 (0.0367)	9.61 (0.0440)	9.61 (0.0370)	10.37(0.0354)	9.19 (0.0307)	9.52 (0.04)
C_2_Cl_2_	8.32 (0.7611)	8.33 (0.7453)	8.82 (0.0549)	8.69 (0.8210)	9.06 (1.6127)	8.26 (0.4736)	8.58 (0.75)
C_2_Br_2_	7.81 (0.9343)	7.81(0.9226)	8.54 (1.0977)	8.09 (1.1650)	8.26 (1.6234)	7.69 (0.7440)	8.03 (1.08)
C_2_F_3_	8.32 (0.1657)	8.34 (0.1700)	8.06 (0.0764)	7.79 (0.0555)	7.98 (0.2056)	8.05 (0.0705)	8.09 (0.12)
C_2_Cl_3_	6.31 (0.1730)	6.33 (0.2409)	6.42 (0.1252)	6.44 (0.2971)	6.37 (0.2740)	6.41 (0.2499)	6.38 (0.26)
C_2_Br_3_	5.90 (0.2702)	5.92 (0.2764)	6.22 (0.2842)	6.22 (0.2373)	6.13 (0.1538)	6.17 (0.2103)	6.09 (0.24)

The present TD-DFT analysis of the UV–visible spectra for the C_2_X_*n*_ (X = F, Cl and Br and *n* = 2–4) species could be useful in both product detection analysis and kinetic studies involving the aforementioned compounds.

### 4.3. Thermochemistry

Standard enthalpies of formation at 298 K for C_2_X_*n*_ species, where X = F, Cl and Br and *n* = 2–4, are of great interest for evaluating the enthalpy changes of their possible decomposition channels. The estimate of this property was performed from isodesmic and isogyric reactions [49,50]. In such a procedure, the reactions used correspond to hypothetical processes in which the number of chemical bonds and the spin multiplicities between reactants and products are conserved. Therefore, the systematic errors arising for both deficiencies in the treatment of the electron correlation energy and incompleteness of the basis sets, are largely cancelled [49,50]. In these calculations, the well-established enthalpies of formation at 298 K for the other species involved in the reactions, listed in the electronic supplementary material, table S7, were employed [51].

The electronic supplementary material, tables S8–S10, show the isodesmic and isogyric reactions used along with the computed enthalpy changes and enthalpies of formation for C_2_X_*n*_, with X = F, Cl and Br and *n* = 2–4, derived from DFT and high-level composite methods. As can be seen, a greater dispersion is observed between the results derived with the DFT formulations (especially those designed for kinetic studies, listed in the electronic supplementary material, tables S11–S13) compared with the composite methods. For that reason, to perform an average between the G3B3//B3LYP/6–311++G(3df,3pd) and G4//B3LYP/6–311++G(3df,3pd) composited methods seems to be the more realistic decision. A summary of these recommended average values derived here, along with those from literature, is presented in table 9. The errors associated with the present determinations were calculated considering the uncertainties in the used quantum-chemical methods (approx. 4.2 kJ mol^−1^), those corresponding to the well-established enthalpies of formation of species employed in the isodesmic reactions (the electronic supplementary material, table S7) and the dispersion of the results. Consequently, a conservative error of ±6.3 kJ mol^−1^ was estimated.

**Table 9.** Enthalpies of formation at 298 K (kJ mol^−1^).

species	Δ*H*_*f*,298_		species	Δ*H*_*f*,298_		species	Δ*H*_*f*,298_	
	this work	literature		this work	literature		this work	literature
C_2_F_4_	−669.6 ± 3.8	−672.8 ± 3.3 [52]	C_2_F_2_	0.6 ± 6.3	5.9 ± 1.3 [52]	C_2_F_3_	−220.9 ± 2.9	—
C_2_Cl_4_	−23.0 ± 4.6	−24.2 ± 4.0 [53]	C_2_Cl_2_	228.1 ± 2.1	226.6 ± 14 [53]	C_2_Cl_3_	230.8 ± 3.8	—
C_2_Br_4_	155.3 ± 5.0	165.4 [21]	C_2_Br_2_	319.6 ± 5.4	318.8 [21]	C_2_Br_3_	375.4 ± 5.9	372.04 [22]
		169.7 [22]			335.1 [20]			
		190.0 [20]						

In the case of the C_2_F_2_, C_2_F_4_, C_2_Cl_2_ and C_2_Cl_4_ species, enthalpy of formation values are reported [52–55]. For C_2_F_2_ and C_2_F_4_, values of 5.9 ± 1.3 and −672.8 ± 3.3 kJ mol^−1^, respectively, were derived from the atomization energy method employing CCSD(T)(FC) geometry optimization with the aug-cc-pVnZ (*n* = D, T, Q, 5–7) basis sets, followed by single point energy calculation at the CCSD(T) level extrapolated to the complete basis set limit [52]. Earlier results for the enthalpy of formation of C_2_F_4_ range between −709.6 ± 8.4 [56] and −653.5 ± 5.4 kJ mol^−1^ [57]. As can be seen from table 9, the present derived result for C_2_F_4_ (−669.6 ± 3.8 kJ mol^−1^) is in very good concordance with the above-mentioned value obtained by Feller *et al*. [52] of −672.8 ± 3.3 kJ mol^−1^, and it also agrees with the dissociation energy reported in the study of the reaction C_2_F_4_ (+M) ⇔ CF_2_ + CF_2_ (+M) [58]. In the case of C_2_F_2_, a slight difference between reported and computed results is found, although both values are fairly similar within the errors of both determinations.

For C_2_Cl_4_, an enthalpy of formation of −24.2 ± 4.0 kJ mol^−1^ was estimated by J.A. Manion as an average of reported experimental determinations mainly from reactions that link Δ*H*_*f*,298_(C_2_Cl_4_) with enthalpies of formation of chlorinated species as CCl_4_ and C_2_HCl_5_, among others [53]. On the other hand, a value with a relatively large associate error, of 226.6 ± 14 kJ mol^−1^, is reported for C_2_Cl_2_, which was computed from an isodesmic reactions approach at the QCISD(T)/6–311+G(2df,p) level of theory [53]. In both cases, the present results of −23.0 ± 4.6 and 228.1 ± 2.1 kJ mol^−1^ are in excellent agreement with previously reported values for the chlorinated closed-shell C_2_Cl_4_ and C_2_Cl_2_ species, respectively, allowing for a reduction of the associated error for the last compound. Furthermore, the estimated enthalpy of formation for C_2_Cl_4_ is in agreement with that arising from the dissociation energy calculated by Gómez *et al*. [14].

For the brominated C_2_Br_*n*_ (*n* = 2–4) species, reported enthalpies of formation are presented without error estimation [20–22,51]. The results derived here are smaller than those previously reported. For C_2_Br_4_, values of 190.0 [20], 165.4 [21] and 169.7 kJ mol^−1^ [22] were predicted at the DK-CCSD(T)/aug-VTZ, G3X and G3B3 levels, respectively. These determinations are higher than the present result of 155.3 ± 5.0 kJ mol^−1^ derived as an average between the G3B3//B3LYP/6–311++G(3df,3pd) and G4//B3LYP/6–311++G(3df,3pd) methods. However, a good concordance with the enthalpy of formation reported by Wang [21] may be accepted if the associated error is considered.

In the case of C_2_Br_2_, Oren *et al*. derived a value of 335.1 kJ mol^−1^ at the DK-CCSD(T)/aug-VTZ level, but they mentioned that it is probably too high by about 2.1 kJ mol^−1^ [20]. Wang reported a different enthalpy of formation of 318.8 kJ mol^−1^ computed at the G3X method [21]. As can be seen from table 9, the result derived here falls within the range of the previous determinations and, again, compares satisfactorily with the estimation at the G3X level. For the C_2_Br_3_ radical, the present value of 375.4 ± 5.9 kJ mol^−1^ is very close to that reported by Burcat *et al*. [22], of 372.04 kJ mol^−1^. A much higher value of 385.4 kJ mol^−1^ is also available in the literature [20]. Additionally, estimations of the errors for the corresponding enthalpies of formation for brominated compounds are also provided.

Finally, accurate enthalpies of formation for the C_2_F_3_ and C_2_Cl_3_ reactive radicals were predicted for the first time, with values of −220.9 ± 2.9 and 230.8 ± 3.8 kJ mol^−1^, respectively.

**Figure 3.**
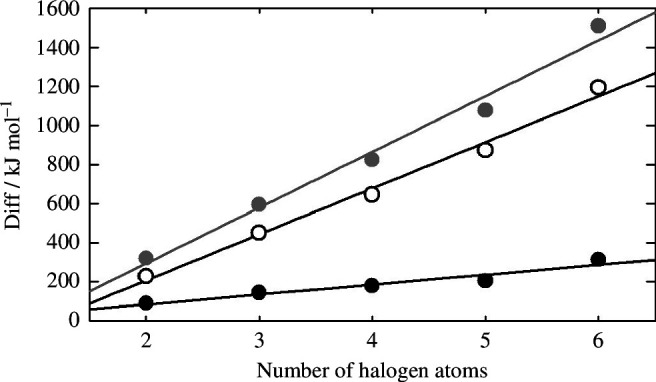
Differences between enthalpies of formation for C_2_X_*n*_ species (X = F, Cl and Br and *n* = 2–6) when (black circles, ●) bromine atoms are replaced by chlorine atoms, (grey circles, ●) bromine atoms are changed by fluorine atoms, and (open circles, ○) chlorine atoms are substituted by fluorine atoms.

Figure 3 depicts the computed differences (Diff) between enthalpies of formation of C_2_X_*n*_ species (X = F, Cl and Br and *n* = 2–6) when bromine atoms are replaced by chlorine or fluorine atoms, and when chlorine atoms are changed by fluorine atoms, as a function of the total number of halogen atoms. For example, Diff = Δ*H*_*f*,298_(C_2_Br_*n*_) - Δ*H*_*f*,298_(C_2_Cl_*n*_) for the first case. The graphic includes enthalpy of formation values derived in this work (table 9), along with the following information for the C_2_X_5_ and C_2_X_6_ species (in kJ mol^−1^): −894.4 ± 2.8 (C_2_F_5_), −21.3 ± 5.4 (C_2_Cl_5_), 183.3 (C_2_Br_5_), −1344.3 ± 3.4 (C_2_F_6_), −148.2 ± 5.7 (C_2_Cl_6_), and 165.5 (C_2_Br_6_) [51]. The very good fit of these representations (with *r*^2^ values better than 0.9) shows that the enthalpies of formation recommended in this work follow the trend observed in the available literature values.

### 4.4. Atmospheric implications

Standard enthalpies of formation previously derived for C_2_X_*n*_ species (X = F, Cl and Br and *n* = 2–4) enable the evaluation of both combustion and atmospheric degradation processes. In the atmospheric context, the C_2_Cl_3_ radical is of particular interest. Reactions of C_2_Cl_3_ with O_2_ [7,8] and NO_2_ [9,59] are well known. In the case of the reaction between the C_2_Cl_3_ radical and O_2_, several exothermic reaction channels were identified. Specifically, the reaction proceeds through a barrierless addition to form the chlorinated vinylperoxy radical, C_2_Cl_3_OO [7,8]. This species can decompose or isomerize to generate various reaction products, such as Cl_2_CO, ClCO, CCl_3_CO, CO and CO_2_, among others [7]. Similarly, four reaction channels were proposed for the C_2_Cl_3_ + NO_2_ reaction, forming products such as C_2_Cl_3_O, CCl_3_CO, CCl_3_, ClCNO, Cl_2_CO, CO and NO [9]. Once again, the formation of a possible reaction intermediate, C_2_Cl_3_NO_2_, followed by isomerization to C_2_Cl_3_ONO, was found in a theoretical study [59].

However, the major degradation process is most likely its oxidation reaction initiated by OH radicals, although the reaction with NO, which reacts rapidly with hydrocarbon radicals [60], may also play a significant role in polluted atmospheres. In these reactions, the formation of an addition adduct is possible, which can lead to the generation of different products. In fact, an analogy can be performed between the reactions of C_2_H_3_ with OH radicals and with NO [61–63], and those corresponding to the C_2_Cl_3_ radical. For this reason, the initial formation of the C_2_Cl_3_OH adduct was considered for the C_2_Cl_3_ + OH reaction. At the G4//B3LYP/6–311++G(3df,3pd) level of theory, employing the total atomization energy (TAE) [64] to estimate Δ*H*_*f*,298_(C_2_Cl_3_OH) = −178.6 kJ mol^−1^ and the value of −37.492 ± 0.026 kJ mol^−1^ for the OH radical [51], a reaction enthalpy of −446.9 kJ mol^−1^ was derived at 298 K for the formation of C_2_Cl_3_OH. Three probable decomposition pathways for this species can yield Cl_2_C=CClO + H, Cl_2_C=O + HCCl and ClCOH + CCl_2_, among others. Additionally, direct Cl atom abstraction from the OH radical to produce C_2_Cl_2_ and ClOH can also be considered, without the formation of an adduct. Reaction enthalpies at 298 K of −157.7, −169.8, −27.8, and −115.0 kJ mol^−1^ were derived, respectively, for the reactions C_2_Cl_3_ + OH → Cl_2_C=CClO + H, C_2_Cl_3_ + OH → Cl_2_C=O + HCCl, C_2_Cl_3_ + OH → ClCOH + CCl_2_ and C_2_Cl_3_ + OH → C_2_Cl_2_ + ClOH, using the following values for enthalpies of formation (in kJ mol^−1^): −107.4 for CCl_2_=CClO (from TAE), 217.997 ± 0.001 for H [51], −220.9 for Cl_2_C=O [51], 319.4 ± 0.8 for HCCl [51], −74.8 ± 1.2 for ClOH [51], 11.1 ± 2.5 for ClCOH [65], and 229.4 ± 0.8 for CCl_2_ [51]. In the case of C_2_Cl_3_ + NO reaction, two possible addition adducts were considered: C_2_Cl_3_NO and C_2_Cl_3_ON. The enthalpies of formation at 298 K for these adducts were estimated to be 136.8 and 299.7 kJ mol^−1^, respectively, from total atomization energies at the G4//B3LYP/6–311++G(3df,3pd) level. These results, along with the enthalpy of formation of 91.090 ± 0.062 kJ mol^−1^ for NO [51], lead to reaction enthalpies at 298 K of −185.1 and −22.2 kJ mol^−1^, indicating greater stability for the C_2_Cl_3_NO adduct. Once again, this adduct may decompose to form Cl_2_C=NCl + CO, Cl_2_C=O + ClCN, among other products. Also, it is possible a reaction to form ClNO and C_2_Cl_2_ through Cl atom abstraction from NO. The corresponding reactions C_2_Cl_3_ + NO → Cl_2_C=NCl + CO, C_2_Cl_3_ + NO → Cl_2_C=O + ClCN and C_2_Cl_3_ + NO → ClNO + C_2_Cl_2_, show reaction enthalpy values at 298 K of −343.0, −413.4 and −39.5 kJ mol^−1^, respectively, calculated employing the following enthalpy of formation values (in kJ mol^−1^): 89.4 for Cl_2_C=NCl (from TAE), −110.523 ± 0.026 for CO [51], −220.9 for Cl_2_C=O [51] and 129.4 kJ mol^−1^ for ClCN (from TAE).

As can be seen, all the processes evaluated here are highly exothermic, suggesting that C_2_Cl_3_ reacts rapidly with both OH and NO. The identification of the possible transition states connecting the adducts to the different products is still required to fully elucidate the reaction mechanisms. Nevertheless, it is clear that barrierless addition reactions leading to the formation of the corresponding adducts result in highly exothermic processes.

## 5. Conclusions

The present study provides accurate structural and thermochemical data of the C_2_X_3_ radicals and the related closed-shell molecules C_2_X_4_ and C_2_X_2_ (with X = F, Cl and Br). These findings also allowed us to validate the employed methods. The computed equilibrium structures and harmonic vibrational frequencies for C_2_X_4_ and C_2_X_2_ exhibited a very good agreement regarding previous literature data for all functionals employed. Notably, the B3LYP method shows the smallest MAD from experimental values. Additionally, a detailed TD-DFT analysis of the UV–visible spectra of the studied species was performed, and the values derived in this study for C_2_X_3_ and C_2_X_2_ represent a reasonable approximation in the absence of experimental information. In the case of C_2_Cl_4_, the computed IR spectrum was compared against the experimental results determined here.

Structural and spectroscopic characterization of the C_2_F_3_, C_2_Cl_3_ and C_2_Br_3_ radicals, along with the estimation of the enthalpies of formation of C_2_F_3_ and C_2_Cl_3_ were presented here for the first time, to our knowledge. Recommended enthalpies of formation at 298 K were derived by averaging results from the G3B3//B3LYP/6–311++G(3df,3pd) and G4//B3LYP/6–311++G(3df,3pd) composited methods. For C_2_F_4_, C_2_F_2_, C_2_Cl_4_ and C_2_Cl_2_, the obtained values of −669.6 ± 3.8, 0.6 ± 6.3, −23.0 ± 4.6 and 228.0 ± 2.1 kJ mol^−1^, show highly satisfactory agreement with previous determinations. In the case of brominated compounds, C_2_Br_4_, C_2_Br_3_ and C_2_Br_2_, enthalpies of formation of 155.3 ± 5.0, 375.4 ± 5.9 and 319.6 ± 5.4 kJ mol^−1^ were calculated, which are lower than those previously reported without error estimation. Finally, accurate enthalpies of formation were computed for the first time, to our knowledge, for the C_2_F_3_ and C_2_Cl_3_ radicals, with values of −220.9 ± 2.9 and 230.8 ± 3.8 kJ mol^−1^, respectively. These data can be helpful in future experimental measurements and theoretical determinations. In that sense, some accessible reaction channels for the reactions of C_2_Cl_3_ with OH radicals and with NO are briefly discussed.

## Data Availability

Dataset files with input matrices for all species used in the work [66]. Supplementary material is available online [67].

## References

[1] Chang WD, Senkan SM. 1989 Detailed chemical kinetic modeling of fuel-rich trichloroethane/oxygen/argon flames. Environ. Sci. Technol. **23**, 442–450. (10.1021/es00181a009)

[2] Taylor PH, Tirey DA, Dellinger B. 1996 A detailed kinetic model of the high-temperature pyrolysis of tetrachloroethene. Combust. Flame **104**, 260–271. (10.1016/0010-2180(95)00117-4)

[3] Kostina SA, Bryukov MG, Shestov AA, Knyazev VD. 2003 Kinetics of the reaction of C_2_Cl_3_ with Cl_2_. J. Phys. Chem. A **107**, 1776–1778. (10.1021/jp027162x)

[4] Dharmarathne NK, Mackie JC, Kennedy EM, Stockenhuber M. 2017 Mechanism and rate of thermal decomposition of hexachlorocyclopentadiene and its importance in PCDD/F formation from the combustion of cyclodiene pesticides. J. Phys. Chem. A **121**, 5871–5883. (10.1021/acs.jpca.7b05209) 28682607

[5] Taylor PH, Tirey DA, Rubey WA, Dellinger B. 1994 Detailed modeling of the pyrolysis of trichloroethene: formation of chlorinated aromatic species. Combust. Sci. Technol. **101**, 75–102. (10.1080/00102209408951867)

[6] Russell JJ, Seetula JA, Gutman D, Senkan SM. 1989 Kinetics of reactions of chlorinated vinyl radicals (CH_2_CCl and C_2_Cl_3_) with molecular oxygen. J. Phys. Chem. **93**, 1934–1938. (10.1021/j100342a047)

[7] Xiang T, Liu K, Zhao S, Su H, Kong F, Wang B. 2007 Multichannel reaction of C_2_Cl_3_ + O_2_ studied by time-resolved Fourier transform infrared emission spectroscopy. J. Phys. Chem. A **111**, 9606–9612. (10.1021/jp074058c) 17705358

[8] Wang H, Li J, Song X, Li Y, Hou H, Wang B, Su H, Kong F. 2006 Computational study of the reaction of chlorinated vinyl radical with molecular oxygen (C_2_Cl_3_ + O_2_). J. Phys. Chem. A **110**, 10336–10344. (10.1021/jp0633345) 16928127

[9] Xiang TC, Liu KH, Su HM. 2007 Experimental study of C_2_Cl_3_+NO_2_ reaction. Chin. J. Chem. Phys. **20**, 407–411. (10.1088/1674-0068/20/04/407-411)

[10] Liu K, Xiang T, Wu W, Zhao S, Su H. 2008 Reaction mechanisms of C_2_Cl_3_ + NO_2_ via nitro and nitrite adducts. J. Phys. Chem. A **112**, 10807–10815. (10.1021/jp8031034) 18837492

[11] Bryukov MG, Kostina SA, Knyazev VD. 2003 Kinetics of the unimolecular decomposition of the C_2_Cl_3_ radical. J. Phys. Chem. A **107**, 6574–6579. (10.1021/jp034205g)

[12] Kumaran SS *et al*. 1997 Experiments and theory on the thermal decomposition of CHCl_3_ and the reactions of CCl_2_. J. Phys. Chem. A **101**, 8653–8661. (10.1021/jp971723g)

[13] Gómez ND, D’Accurso V, Freytes VM, Manzano FA, Codnia J, Azcárate ML. 2013 Kinetic study of the CCl_2_ radical recombination reaction by laser‐induced fluorescence technique. Int. J. Chem. Kinet. **45**, 306–313. (10.1002/kin.20766)

[14] Gómez ND, Codnia J, Azcárate ML, Cobos CJ. 2017 Quantum chemical and kinetic study of the CCl_2_ self-recombination reaction. Comput. Theor. Chem. **1121**, 1–10. (10.1016/j.comptc.2017.10.004)

[15] Heicklen J. 1966 Photolysis of trifluoroethylene iodide in the presence of nitric oxide and oxygen. J. Phys. Chem. **70**, 618–627. (10.1021/j100875a003)

[16] Wurfel BE, Pugliano N, Bradforth SE, Saykally RJ, Pimentel GC. 1991 Broadband transient infrared laser spectroscopy of trifluorovinyl radical C_2_F_3_ : experimental and ab initio results. J. Phys. Chem. **95**, 2932–2937. (10.1021/j100160a052)

[17] Wurfel BE, Thoma A, Bondybey VE. 1992 Vibrational spectroscopy of C_2_F_3_ in an Ar matrix. Chem. Phys. Lett. **198**, 135–142. (10.1016/0009-2614(92)90061-Q)

[18] Cobos CJ, Tellbach E, Troe J. 2014 Shock wave study of the thermal dissociations of C_3_F_6_ and c-C_3_F_6_. I. Dissociation of hexafluoropropene. J. Phys. Chem. A **118**, 4880–4888. (10.1021/jp501569a) 24905383

[19] Kong Q, Wulff M, Lee JH, Bratos S, Ihee H. 2007 Photochemical reaction pathways of carbon tetrabromide in solution probed by picosecond X-ray diffraction. J. Am. Chem. Soc. **129**, 13584–13591. (10.1021/ja073503e) 17939658

[20] Oren M, Iron MA, Burcat A, Martin JML. 2004 Thermodynamic Properties of C_1_ and C_2_ Bromo Compounds and Radicals. A Relativistic ab Initio StudyClick to copy article link. J. Phys. Chem. A **108**, 7752–7761. (10.1021/jp0475786)

[21] Wang L. 2008 Theoretical studies on the thermochemistry of stable closed-shell C1 and C2 brominated hydrocarbons. J. Phys. Chem. A **112**, 4951–4957. (10.1021/jp0774443) 18473445

[22] Burcat A, Khachatryan L, Dellinger B. 2009 Thermochemistry of chlorine‐containing hydrocarbons related to waste combustion. Int. J. Chem. Kinet. **41**, 113–122. (10.1002/kin.20379)

[23] Becke AD. 1993 Density functional thermochemistry. III. The role of exact exchange. J. Chem. Phys. **98**, 5648–5652. (10.1063/1.464913)

[24] Xu X, Goddard WA III. 2004 From the cover: the X3LYP extended density functional for accurate descriptions of nonbond interactions, spin states, and thermochemical properties. Proc. Natl Acad. Sci. USA **101**, 2673–2677. (10.1073/pnas.0308730100) 14981235 PMC374194

[25] Boese AD, Martin JML. 2004 Development of density functionals for thermochemical kinetics. J. Chem. Phys. **121**, 3405–3416. (10.1063/1.1774975) 15303903

[26] Zhao Y, Truhlar DG. 2008 The M06 suite of density functionals for main group thermochemistry, thermochemical kinetics, noncovalent interactions, excited states, and transition elements: two new functionals and systematic testing of four m06-class functionals and 12 other functionals. Theor. Chem. Acc. **120**, 215–241. (10.1007/s00214-007-0310-x)

[27] Yu HS, He X, Li SL, Truhlar DG. 2016 MN15: a Kohn-Sham global-hybrid exchange-correlation density functional with broad accuracy for multi-reference and single-reference systems and noncovalent interactions. Chem. Sci. **7**, 5032–5051. (10.1039/c6sc00705h) 30155154 PMC6018516

[28] Zhao Y, Truhlar DG. 2008 Exploring the limit of accuracy of the global hybrid meta density functional for main-group thermochemistry, kinetics, and noncovalent interactions. J. Chem. Theory Comput. **4**, 1849–1868. (10.1021/ct800246v) 26620329

[29] Frisch MJ *et al*. 2016 Gaussian 16, revision c.01. Wallingford, CT: Gaussian, Inc.

[30] Baboul AG, Curtiss LA, Redfern PC, Raghavachari KJ. 1999 Gaussian-3 theory using density functional geometries and zero-point energies. J. Chem. Phys. **110**, 7650–7657. (10.1063/1.478676)

[31] Curtiss LA, Redfern PC, Raghavachari K. 2007 Gaussian-4 theory. J. Chem. Phys. **126**, 084108–084112. (10.1063/1.2436888) 17343441

[32] Hirata S, Head-Gordon M. 1999 Time-dependent density functional theory for radicals: an improved description of excited states with substantial double excitation character. Chem. Phys. Lett. **302**, 375–382. (10.1016/S0009-2614(99)00137-2)

[33] Hellwege KH, Hellwege AM (eds). 1976 Landolt-Bornstein: group ii: atomic and molecular physics volume 7: structure data of free polyatomic molecules. Berlin, Germany: Springer-Verlag.

[34] Strand TG. 1967 Electron diffraction investigation of and hexabromobenzene. Acta Chem. Scand. **21**, 1033–1045. (10.3891/acta.chem.scand.21-1033)

[35] Kuchitsu K (ed). 1995 Landolt-Bornstein: group ii: molecules and radicals volume 23: structure data for free polyatomic molecules: supplement to ii/7, ii/15 and ii/21. Berlin Heidelberg, Germany: Springer.

[36] Chau FT, Yuen ML. 1996 Determination of bond lengths of polyatomic species using vibrational frequencies. J. Electron Spectros. Relat. Phenomena **77**, 183–196. (10.1016/0368-2048(95)02499-9)

[37] De Alti G, Galasso V, Costa G. 1965 Potential energy constants, mean-square amplitudes of vibration and rotational distortion constants for C_2_F_4_, C_2_Cl_4_ and C_2_Br_4_. Spectrochim. Acta **21**, 649–658. (10.1016/0371-1951(65)80021-2)

[38] Shimanouchi T. 1972 Tables of molecular vibrational frequencies consolidated volume I, pp. 1–160. NBS National Standard Reference Data Series (NSRDS): U.S. Government, Commerce Department, National Institute of Standards and Technology (NIST), Executive Agency Publications.

[39] Bürger H, Schneider W, Sommer S, Thiel W, Willner H. 1991 The vibrational spectrum and rotational constants of difluoroethyne FC 3/4 CF. Matrix and high resolution infrared studies and ab initio calculations. J. Chem. Phys. **95**, 5660–5669. (10.1063/1.461640)

[40] McNaughton D, Elmes P. 1992 High resolution Fourier transform infrared spectrum of difluoroethyne, FCCF. Spectrochim. Acta **48**, 605–611. (10.1016/0584-8539(92)80051-W)

[41] Shimanouchi T. 1977 Tables of molecular vibrational frequencies. Consolidated volume II. J. Phys. Chem. Ref. Data **6**, 993–1102. (10.1063/1.555560)

[42] Linstrom PJ, Mallard WG (eds). 2023 NIST chemistry webbook, NIST standard reference database number 69. Gaithersburg, MD: National Institute of Standards and Technology. (10.18434/T4D303)

[43] Sharpe S, Hartnett B, Sethi HS, Sethi DS. 1987 Absorption cross-sections of CF_2_ in the Ã ^1^B_1_- ^1^A_1_ transition at 0.5 nm intervals and absolute rate constant for 2CF_2_→C_2_F_4_ at 298 ± 3 K. J. Photochem. **38**, 1–13. (10.1016/0047-2670(87)87001-6)

[44] Eden S, Barc B, Mason NJ, Hoffmann SV, Nunes Y, Limão-Vieira P. 2009 Electronic state spectroscopy of C_2_Cl_4_. Chem. Phys. **365**, 150–157. (10.1016/j.chemphys.2009.10.010)

[45] Berry MJ. 1974 Chloroethylene photochemical lasers: vibrational energy content of the HCl molecular elimination products. J. Chem. Phys. **61**, 3114–3143. (10.1063/1.1682468)

[46] Laguer JR, Hummel LE, Bohmfalk EF, Park JD. 1950 The near ultraviolet absorption spectra of some fluorinated derivatives of methane and ethylene. J. Am. Chem. Soc. **72**, 5486–5489. (10.1021/ja01168a029)

[47] UV-Vis spectral databases. John Wiley & Sons. See https://spectrabase.com/spectrum/7lmzDf6MU0X.

[48] Gupta D, Choi H, Kwon DC, Yoon JS, Antony B, Song MY. 2017 Cross sections for electron collision with difluoroacetylene. J. Phys. B At. Mol. Opt. Phys. **50**, 085202. (10.1088/1361-6455/aa6325)

[49] Hehre WJ, Radom L, Schleyer PVR, Pople JA. 1986 Ab initio molecular orbital theory. New York, NY: Wiley.

[50] Berry RJ, Burgess DRF, Nyden MR, Zachariah MR, Schwartz M. 1995 Halon thermochemistry: Ab initio calculations of the enthalpies of formation of fluoromethanes. J. Phys. Chem. **99**, 17145–17150. (10.1021/j100047a017)

[51] Burkholder JB *et al*. 2019 Chemical kinetics and photochemical data for use in atmospheric studies, evaluation no. 19. Pasadena: JPL Publication 19-5, Jet Propulsion Laboratory. See http://jpldataeval.jpl.nasa.gov.

[52] Feller D, Peterson KA, Dixon DA. 2011 Ab initio coupled cluster determination of the heats of formation of C_2_H_2_F_2_, C_2_F_2_, and C_2_F_4_. J. Phys. Chem. A **115**, 1440–1451. (10.1021/jp111644h) 21306144

[53] Manion JA. 2002 Evaluated enthalpies of formation of the stable closed shell C_1_ and C_2_ chlorinated hydrocarbons. J. Phys. Chem. Ref. Data **31**, 123–172. (10.1063/1.1420703)

[54] Pedley JB. 1994 Thermochemical data and structures of organic compounds. College Station, TX: Thermodynamics Data Center.

[55] Baranovskii VI, Skorobogatov GA. 2016 Quantum-chemical simulation of the gas-phase molecular, thermodynamic, and kinetic parameters of CF, CF_2_, and CF_3_ radicals and CF_4_, C_2_F_2_, C_2_F_4_, and C_2_F_6_ molecules. Russ. J. Gen. Chem. **86**, 241–250. (10.1134/S1070363216020067)

[56] v. Wartenberg H, Schiefer J. 1955 Bildungswärmen von fluor‐chlor‐kohlenstoff‐verbindungen. Z. Anorg. Allg. Chem. **278**, 326–332. (10.1002/zaac.19552780514)

[57] Neugebauer CA, Margrave JL. 1956 The heats of formation of tetrafiuoroethylene, tetrafluoromethane and, l, l-difluoroethylene. J. Phys. Chem. **60**, 1318–1321. (10.1021/j150543a039)

[58] Cobos CJ, Croce AE, Luther K, Sölter L, Tellbach E, Troe J. 2013 Experimental and modeling study of the reaction C_2_F_4_ (+ M) ⇔ CF_2_ + CF_2_ (+ M). J. Phys. Chem. A **117**, 11420–11429. (10.1021/jp408363s) 24106788

[59] Li Y, Liu H ling, Huang X ri, Wang D quan, Sun C chung, Tang A chin. 2009 Theoretical study on the mechanism of C_2_Cl_3_ + NO_2_ reaction. Theor. Chem. Acc. **123**, 431–441. (10.1007/s00214-009-0549-5)

[60] Striebel F, Jusinski LE, Fahr A, Halpern JB, Klippenstein SJ, Taatjes CA. 2004 Kinetics of the reaction of vinyl radicals with NO: ab initio theory, master equation predictions, and laser absorption measurements. Phys. Chem. Chem. Phys. **6**, 2216–2223. (10.1039/B401163E)

[61] Benson SW. 1994 Kinetics and thermochemistry of the reaction of acetylene and nitric oxide. Int. J. Chem. Kinet. **26**, 997–1011. (10.1002/kin.550261005)

[62] Knyazev VD. 2017 Kinetics and mechanism of the reaction of recombination of vinyl and hydroxyl radicals. Chem. Phys. Lett. **685**, 165–170. (10.1016/j.cplett.2017.07.040)

[63] Zou P, Klippenstein SJ, Osborn DL. 2005 The vinyl + NO reaction: determining the products with time-resolved Fourier transform spectroscopy. J. Phys. Chem. A **109**, 4921–4929. (10.1021/jp050093c) 16833839

[64] Ochterski JW. 2000 Thermochemistry in Gaussian. Gauss. Inc. **1**, 1–19. http://www.gaussian.com/g_whitepap/thermo.htm

[65] Ruscic B, Bross DH. 2023 Active thermochemical tables (ATcT) values based on ver. 1.202 of the thermochemical network. Lemont, IL: Argonne National Laboratory. See http://ATcT.anl.gov.

[66] Badenes M, Ferreira ML, Dubois FI, Tucceri ME, Badenes M. 2024 Molecular, spectroscopic and thermochemical characterization of C_2_Cl_3_, C_2_F_3_ and C_2_Br_3_ radicals and related species. Dryad Digital Repository. (10.5061/dryad.pc866t1xs) PMC11576102 39569350

[67] Ferreira ML, Dubois FI, Tucceri ME, Badenes MP. 2024. Data from: Molecular, spectroscopic and thermochemical characterization of C_2_Cl_3_, C_2_F_3_ and C_2_Br_3_ radicals and related species. Figshare. (10.6084/m9.figshare.c.7539158) PMC11576102 39569350

